# A deep learning method for optimizing semantic segmentation accuracy of remote sensing images based on improved UNet

**DOI:** 10.1038/s41598-023-34379-2

**Published:** 2023-05-10

**Authors:** Xiaolei Wang, Zirong Hu, Shouhai Shi, Mei Hou, Lei Xu, Xiang Zhang

**Affiliations:** 1 The School of Geoscience and Technology, Zhengzhou University, Zhengzhou, 450001 China; 2 Joint Laboratory of Eco-Meteorology, Zhengzhou University, Chinese Academy of Meteorological Sciences, Zhengzhou University, Zhengzhou, 450000 China; 3 National Engineering Research Center for Geographic Information System, School of Geography and Information Engineering, China University of Geosciences (Wuhan), Wuhan, 430074 China; 4 SongShan Laboratory, Zhengzhou, 450046 China

**Keywords:** Environmental sciences, Environmental social sciences

## Abstract

Semantic segmentation of remote sensing imagery (RSI) is critical in many domains due to the diverse landscapes and different sizes of geo-objects that RSI contains, making semantic segmentation challenging. In this paper, a convolutional network, named Adaptive Feature Fusion UNet (AFF-UNet), is proposed to optimize the semantic segmentation performance. The model has three key aspects: (1) dense skip connections architecture and an adaptive feature fusion module that adaptively weighs different levels of feature maps to achieve adaptive feature fusion, (2) a channel attention convolution block that obtains the relationship between different channels using a tailored configuration, and (3) a spatial attention module that obtains the relationship between different positions. AFF-UNet was evaluated on two public RSI datasets and was quantitatively and qualitatively compared with other models. Results from the Potsdam dataset showed that the proposed model achieved an increase of 1.09% over DeepLabv3 + in terms of the average F1 score and a 0.99% improvement in overall accuracy. The visual qualitative results also demonstrated a reduction in confusion of object classes, better performance in segmenting different sizes of object classes, and better object integrity. Therefore, the proposed AFF-UNet model optimizes the accuracy of RSI semantic segmentation.

## Introduction

The semantic segmentation models of remote sensing imagery (RSI) can achieve pixel-level object classifications. Therefore, the classifications obtained by semantic segmentation can meet the demands of precise object monitoring in application fields such as urban land management^1–4^, environmental protection^5^, and natural resource monitoring^6^. With the application of some creative works (i.e. Relu^7^, Dropout^8^, Batch Norm^9^, and ResNet^10^), Convolution Neural Network (CNN) has had much success in computer vision. CNN is gradually applied to the RSI semantic segmentation task to improve the accuracy of object extractions. CNN-based segmentation models typically obtain semantic representations using stacked convolutions and pooling operations, and they restore the image size by upsampling. A common design used in CNN-based semantic segmentation models is the encoder–decoder structure.

Various encoder–decoder designs^11–14^ have been applied for semantic segmentation tasks, and their development remains stalled due to two challenges: (1) There are different sizes of objects on RSI, and the algorithm needs to take the segmentation accuracy of them into account simultaneously; and (2) the coverage of RSI is large, and several easily confused geo-objects exist.

In RSI semantic segmentation, small-size objects and large-size objects typically exist in one patch, and the models need to segment the different sizes of objects simultaneously. This requires that the model possesses strong context aggregation capabilities and processing capabilities for multi-scale features. The semantic information of objects of different sizes exists in different levels of layers of the model. This means that fusing different levels of features directly may cause negative interference between the semantic information of objects of different sizes. A potential solution is using the attention mechanism to enable the model to automatically select what level of features is to be focused on according to the content of the input image during the feature fusion. To address this issue, the dense skip connection (DSC) architecture and adaptive fusion attention module (AFAM) are proposed. By using this architecture and module, the corresponding weights can be automatically assigned to different levels of features in the process of deep and shallow information fusion.

The existence of confused object classes in RSI requires that segmentation models must be capable of learning accurate feature representations. The semantic segmentation results are generated by considering a large area as a whole instead of precisely classifying each pixel^15^. Therefore, inaccurate feature representations can lead to severe mis-segmentation. To obtain accurate feature representations of confused object classes, attention mechanisms are typically used to overcome the limitation of local dependencies in neural networks. The attention mechanism can consider the relationship of features from the channel and position aspect and generate an attention weight map to selectively suppress or enhance features. The tailored channel attention convolution block (CACB) and a spatial attention module (SAM) are applied in the proposed model. These enable the model to obtain accurate feature representations, thereby reducing the class confusion.

The primary contributions of this article are as follows:

- The DSC structure and AFAM are proposed to fuse different levels of features, and this can obtain the correlation of different levels of the feature blocks to improve the segmentation of different sizes of object class (e.g. the buildings and vehicles class).
- The CACB and SAM are applied to improve the accuracy of confused object classes (e.g. the background class and non-background class) and the integrity of objects (especially the segmentation of buildings in Potsdam dataset).
- The effectiveness of the proposed model is verified by using quantitative and qualitative experiments.

## Related works

### Encoder-decoder architecture

Encoder-decoder models are a representative family of neural networks that show better performance in dense prediction tasks. UNet^14^ is proposed for the medical image segmentation task, which is a typical decoder-encoder structure. The decoder-encoder structure is also widely used in image generation^16^ and object detection^17^. Some models have been developed, such as SegNet^13^, RefineNet^18^ and LW-RefineNet^19^. Many encoder-decoder models aim to improve performance by expanding the receptive field, such as PSPNet^12^ and DeepLabV3 + ^11^. To boost the ability to gather global in-formation, PSPNet employs a PPM (pyramid pooling module). DeepLabV3 + inherits the atrous convolution used in DeepLabV1-DeepLabV3^20–22^. intending to expand the receptive field. To improve model performance, the author proposed a lightweight decoder-encoder structure model^23^.

One of the limitations of the related work is their insufficient fusion of deep and shallow features. Hence, these models have a limited contribution to improving the se-mantic segmentation accuracy. The proposed model in this paper (AFF-UNet) addresses the above limitation using DSC and attention mechanisms.

### Skip connection

Fully convolutional networks (FCN)^24^ was the first model to apply CNN to semantic segmentation tasks, and many subsequent models have been designed based on it. Skip connections are used in FCN to fuse the local and the global information. The skip connections (referred to as “shortcut connections”) in ResNet^10^ are used to solve gradient explosion and gradient vanishing. The authors of DenseNet^25^ used dense connections to guarantee that each layer in the designed dense block uses all of the preceding features as input. The related research showed that the use of skip connections leads to more accurate feature representations, thereby improving the performance. The proposed model is an encoder–decoder structure with the use of DSC due to the effectiveness of skip connections.

### Attention mechanism

In the computer vision domain, the attention mechanism is commonly employed. Its essential function is to judge which part of the information is more important and choose to enhance or suppress it, such SENet^26^, DANet^27^, CBAM^28^ and BAM^29^. Taking DANet as an example, it created a position attention component for learning the spatial interrelations and a channel attention component for modelling channel interrelations. The self-attention mechanism^30^ is another widely used model, mainly including the Swin Transformer model and its derivative works. The Swin Transformer^31^ is a combination of self-attention and more visual prior knowledge. Its greatest contribution is the introduction of shifted windows, which is essentially an application of inductive bias to visual tasks. Multi-scale features are crucial for visual tasks, and both the Swin Transformer and the AFF-UNet attach great importance to this. They have many similarities in their model structure design, such as increasing the number of feature channels when the image size decreases, which generates multi-scale features. Attention mechanisms have also been employed in RSI segmentation^32–34^.This paper describes (1) the design of a novel attention module called AFAM. It is characterized by emphasizing the correlation of the feature blocks rather than the feature channels; and (2) the tailored CACB and SAM are applied to obtain more accurate feature representations.

### Remote sensing image semantic segmentation

Since CNN has caused many breakthroughs in computer vision tasks of natural images, it has been widely employed in the work of the semantic segmentation of RSI^35^.Some researchers have used CNN for some specific applications on RSI. These tasks have included but have not been limited to the extraction of multiple classes of geo-objects in the image, as in this paper, but also the extraction of only a single class of geo-objects, such as building extraction^36,37^, road extraction^38–40^, cloud and snow detection^41^, and urban village mapping^42^. Some models were developed, such as AWNet^43^, HA-MPPNet^44^ and HED-UNet^45^. The authors explored the combination of FCN, UNet, and LSTM in parallel for the RSI segmentation task^46^. The authors used pre-trained deep learning model DeepLabv3 + for land use and land cover classification on RSI^47^.

In general, researchers have made many attempts to optimize the segmentation accuracy of RSI. However, RSI semantic segmentation remains a challenging task. The focus of this paper is not only to fuse the context information but also to realize the automatic assignment of weights of different levels feature blocks.

## Methods

**Figure 1:**
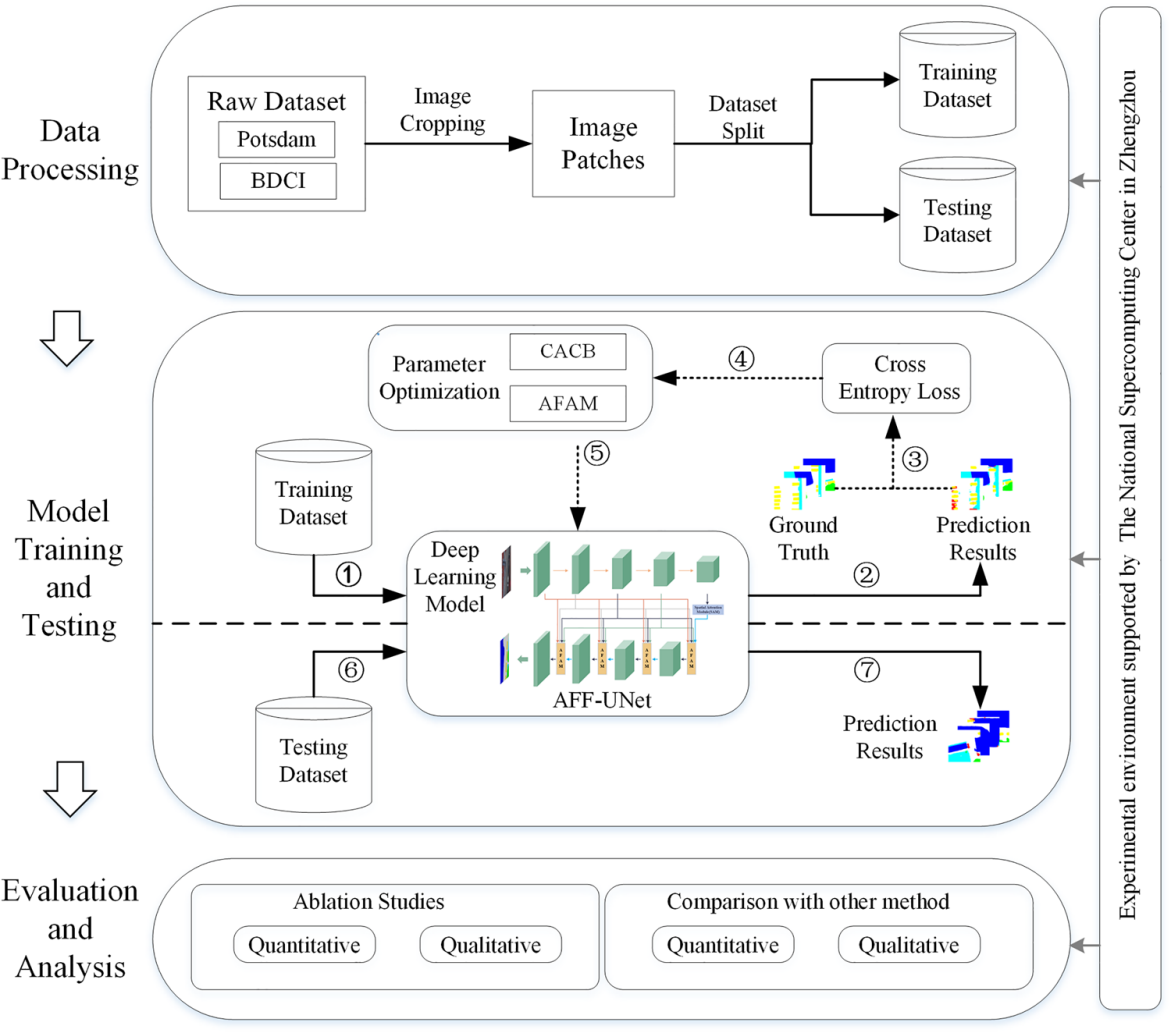
Workflow of the paper.

The workflow of this paper consists of three main parts (Fig. 1): the first part is data preprocessing, which mainly includes image cropping and dataset split. The second part is the model training and testing. The last part is the evaluation and analysis of the experimental results. The diagram shows the correspondence between the various parts of the workflow. All experiments in this paper were performed on the National Supercomputing Center in Zhengzhou.

**Figure 2:**
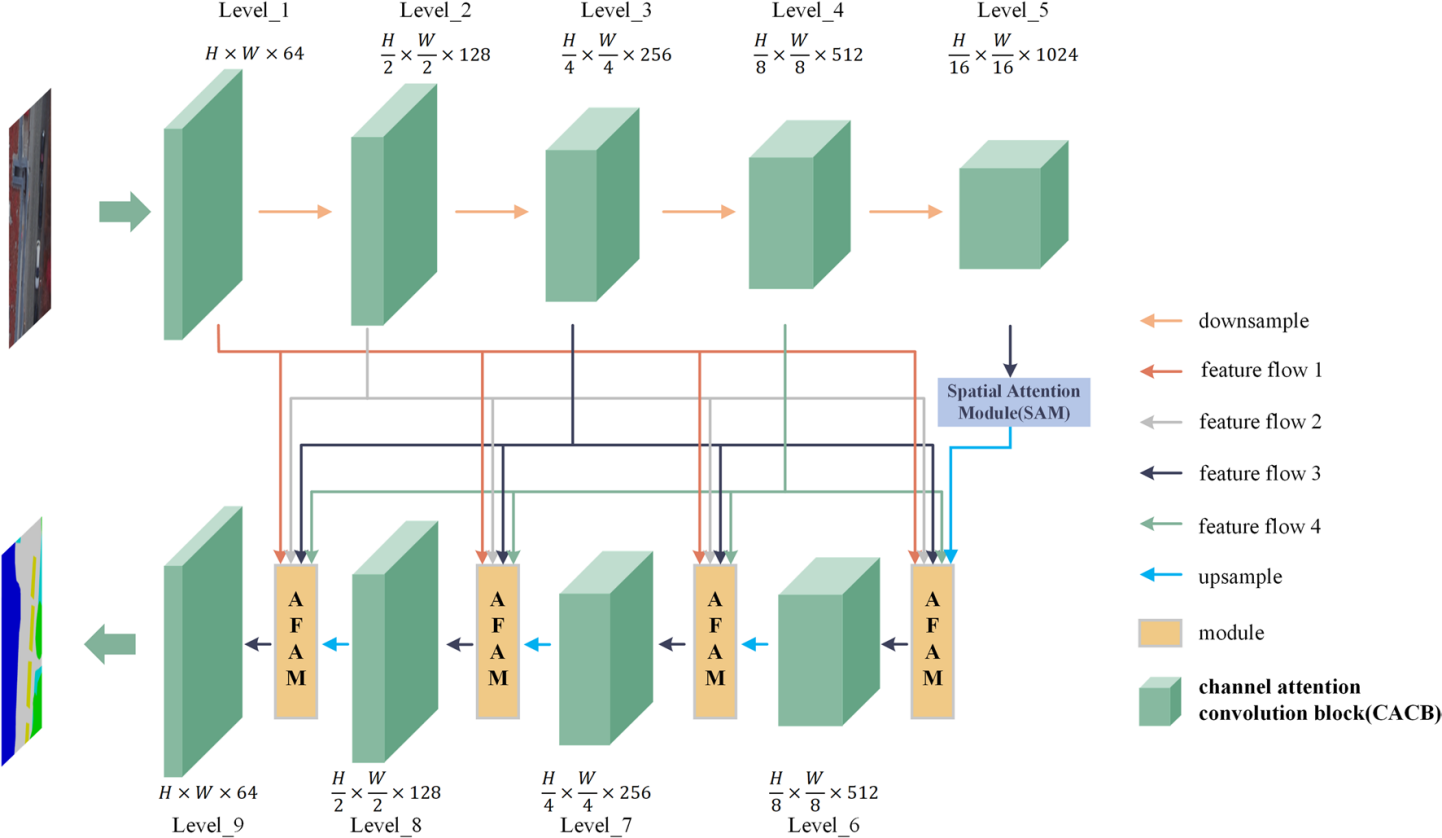
Overview of the proposed AFF-UNet architecture for RSI semantic segmentation.

In this paper, an RSI semantic segmentation model called the Adaptive Feature Fusion UNet (AFF-UNet) is proposed (shown in Fig. 2). The proposed model is developed from UNet, and includes three parts: 1. DSC and AFAM were designed to make the model have a stronger context aggregation ability and could adaptively emphasize or suppress different levels of feature blocks instead of feature channels during fusion; 2. CACB was applied to obtain the relationship between the different channels; and 3. SAM was applied to obtain the relationship between the different positions. Details are as follows:

1. The feature maps of the first, second, third, and fourth levels in the encoder are directly connected to the adaptive fusion attention module (AFAM) in the decoder through a dense connection structure (consisting of feature flow 1, feature flow 2, feature flow 3, and feature flow 4). The dense skip connections (DSC) improves the feature fusion ability and feature utilization of the model, which in turn allows the model to obtain more accurate feature representations and reduce the misidentification of confused classes.
2. The AFAM can differentiate between features from different levels and assign weights to these features to improve the segmentation of different-sized objects. The input of the AFAM comes from both the encoder and the decoder feature maps. The module primarily performs feature map fusion and assigns weights to the input's five feature map blocks to improve the segmentation of different-sized categories.
3. The Channel Attention Convolution Block (CACB) consists of convolution, batch normalization, activation function, and compress-activation operations. It does not change the size of the feature map but alters the number of channels in the feature map. In this paper, the model’s input is 3 or 4 channels, and the encoder’s channel changes as follows: 3 or 4 channels, 64 channels, 128 channels, 256 channels, 512 channels, 1024 channels, accompanied by a decrease in the size of the feature map. The decoder's channel changes are 512 channels, 256 channels, 128 channels, and 64 channels, accompanied by an increase in the size of the feature map.
4. The Spatial Attention Module (SAM) is placed in the middle of the encoder and decoder, it and does not change the size or channels in the feature map. It primarily focuses on the position information of the feature map.

### DSC architecture and the AFAM

**Figure 3:**
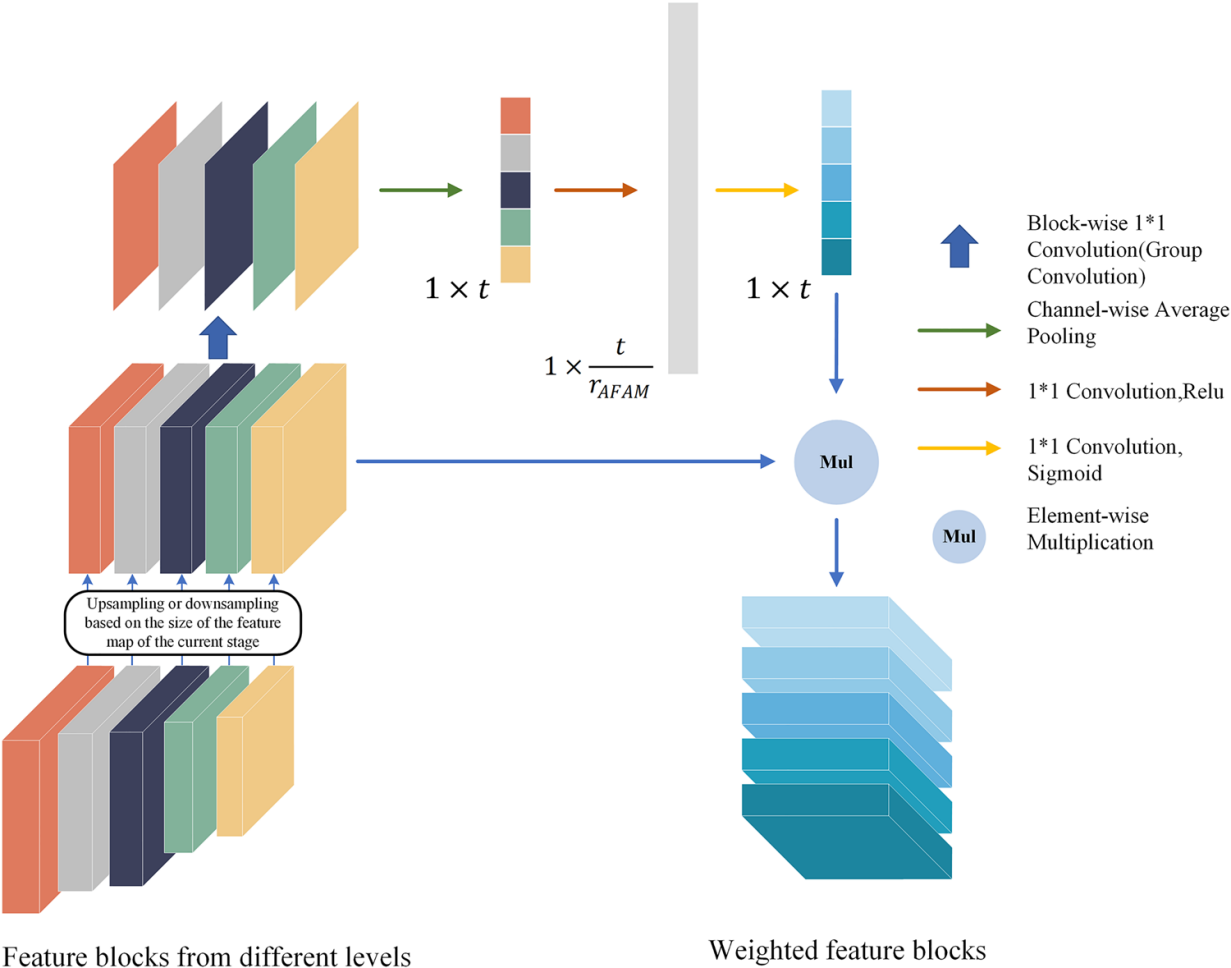
Detailed composition of the designed AFAM.

To enhance the feature fusion ability, the proposed model applies the DSC structure that makes that the information of each level in the encoder be connected to each level in the decoder. The motivation is because it is difficult to predict, which context information to aggregate, and this is more conducive for improving model accuracy. Hence, we used DSC to make the model have the potential to fuse contextual information at any stage. We then let the model automatically select which levels of information should be “paid more attention” to by AFAM (Fig. 3). Unlike the sparse skip connections in the original UNet, the DSC enables the four-level feature maps of the encoder to be directly embedded into each stage of the decoder.

The characteristics of AFAM includes two aspects:

1. Reasonable allocation of feature block-wise weights: Feature maps from the same level have certain commonalities (for example, they are at the same depth and the same theoretical receptive field). AFAM can learn the correlation of the feature blocks from the different levels and assign weights. (i.e. in the AFF-UNet, five levels of feature blocks need to be concatenated, and then five weights are obtained by the AFAM). The Squeeze-and-Excitation (SE) operation only assigns the channel-wise weights of the feature maps. In other words, only the channel-wise correlation between the feature maps is considered, and the correlation between the different levels of feature blocks is not considered. Regarding the above issue, weights (block-wise) to the feature blocks can be assigned using AFAM.
2. Reduce information redundancy caused by DSC: The DSC may have redundant in-formation compared to the sparse skip connections. AFAM can handle possible redundancy by automatically assigning weights to the different levels of features.

The AFAM is a computational unit which can be regarded as nonlinear mapping from the input $\mathrm{X}\in {\mathrm{R}}^{{H}^{^{\prime}}\times {W}^{^{\prime}}\times {C}^{^{\prime}}}$ to the output $\mathrm{O}\in {\mathrm{R}}^{H\times W\times C}$.Here $\mathrm{X}=[{\mathrm{x}}_{B1},{\mathrm{x}}_{B2},\cdots ,{\mathrm{x}}_{Bt}]$, ${\mathrm{x}}_{Bi}\in {\mathrm{R}}^{{H}_{i}\times {W}_{i}\times {C}_{i}}$, Where ${\mathrm{x}}_{Bi}$ represents the i-th feature block, the number of feature blocks $\mathrm{t}=5$, and the number of channels of each feature block is represented as ${C}_{i}$. The first step is to make the size of all feature blocks the same as the size of the next adjacent CACB by up-sampling or down-sampling. Then the dimensionality of each feature block is reduced to 1 channel by $1\times 1$ convolution:

1 $${\mathrm{x}}_{Bi}^{^{\prime}}={\mathrm{x}}_{Bi}{H}_{i},$$

where ${H}_{i}$ represents $1\times 1$ convolution operation, here ${\mathrm{x}}_{Bi}^{^{\prime}}\in {\mathrm{R}}^{H\times W\times 1}$, that is, the above operation reduces the dimensionality of each feature block to 1 channel. Next step is global average pooling:

2 $${\mathrm{z}}_{Bi}=\frac{1}{H\times W}\sum_{m=1}^{H}\sum_{n=1}^{W}{\mathrm{x}}_{Bi}^{^{\prime}}(m,n),$$

where ${\mathrm{z}}_{Bi}$ represents the result obtained by global average pooling of ${\mathrm{x}}_{Bi}^{^{\prime}}$,here $\mathrm{z}=[{\mathrm{z}}_{B1},{\mathrm{z}}_{B2},\cdots ,{\mathrm{z}}_{Bt}]$, z is a $1\times 5$ vector, the next step is to obtain the weight of ${\mathrm{s}}_{Bi}$ of each feature block with the fully-connected layers and the activation function:

3 $$\mathrm{s}=\upsigma \left({W}_{2}\updelta \left({W}_{1}\mathrm{z}\right)\right),$$

where $\mathrm{s}=[{\mathrm{s}}_{B1},{\mathrm{s}}_{B2},\cdots ,{\mathrm{s}}_{Bt}]$, $\upsigma$ denotes Sigmoid, $\updelta$ denotes Relu(the excitation operator of AFAM); ${\mathrm{W}}_{1}\in {R}^{\frac{t}{{r}_{AFAM}}\times t}$, ${\mathrm{W}}_{1}$ denotes the $1\times 1$ convolution with the ratio ${r}_{AFAM}$, we applied ${r}_{AFAM}=0.25$ in this article; ${\mathrm{W}}_{2}\in {R}^{t\times \frac{t}{{r}_{AFAM}}}$, denotes the $1\times 1$ convolution that restores the dimension to the same as the numbers t of feature blocks. Then the weight ${\mathrm{s}}_{Bi}$ of each feature block is obtained after Sigmoid function. The final step is elementwise-multiplication between each feature block ${\mathrm{x}}_{Bi}$ and corresponding weight ${\mathrm{s}}_{Bi}$:

4 $${\widetilde{\mathrm{x}}}_{Bi}={\mathrm{x}}_{Bi}{\mathrm{s}}_{Bi}.$$

The output of AFAM is $\mathrm{O}=[{\widetilde{\mathrm{x}}}_{B1},{\widetilde{\mathrm{x}}}_{B2},\cdots ,{\widetilde{\mathrm{x}}}_{Bt}]$.

### Channel attention convolution block (CACB)

**Figure 4:**
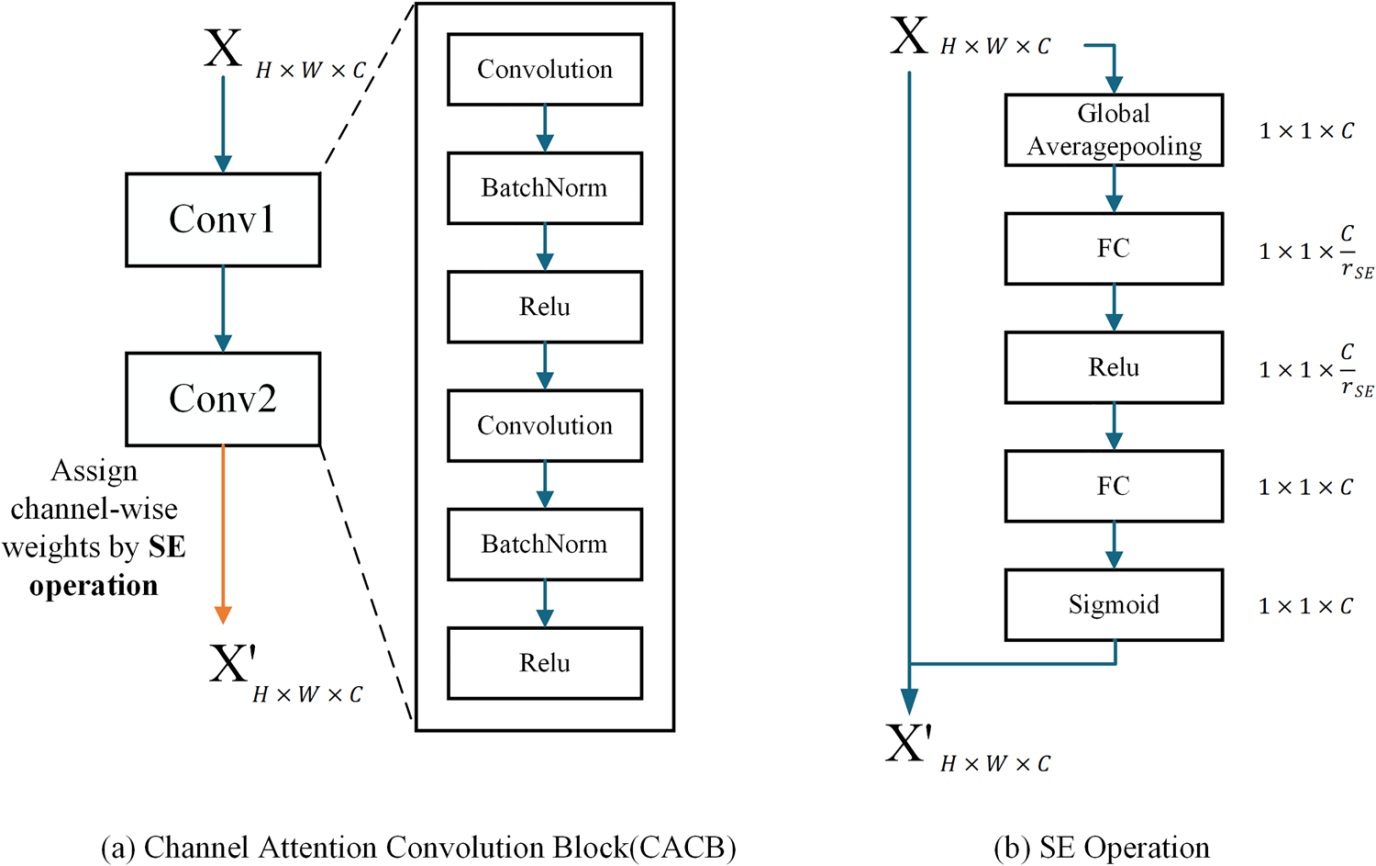
Detailed composition of the designed CACB and SE operation.

The CACB (Fig. 4a) contains two sets of combination (convolution, batch normalization, Relu activation) operations and a SE operation. The SE operation (Fig. 4b) includes two steps: Squeeze and Excitation. Squeeze compresses the features of the H × W × C into 1 × 1 × C through the squeeze operator, that is, compresses each channel into a number. The Excitation operation obtains the weight of each channel using two convolution-activation operations (the second activation function is called the excitation operator) and finally applies the obtained weight to the corresponding channel, that is, reweight. The difference between AFAM and the SE Operation is that the SE assigns weights to each feature channel, while AFAM assigns weights to different levels of feature blocks. CACB can automatically obtain the importance of each feature channel for segmentation and then choose to enhance or suppress each feature channel. ${r}_{SE}$ is a hyperparameter that allows us to vary the capacity of the SE operation. In the proposed model, ${r}_{SE}$ was set to two, which is the optimal configuration obtained by experiments. Following BAM^29^, we chose the global average pooling as the squeeze operator and Sigmoid as the excitation operator.

### Spatial attention module (SAM)

**Figure 5:**
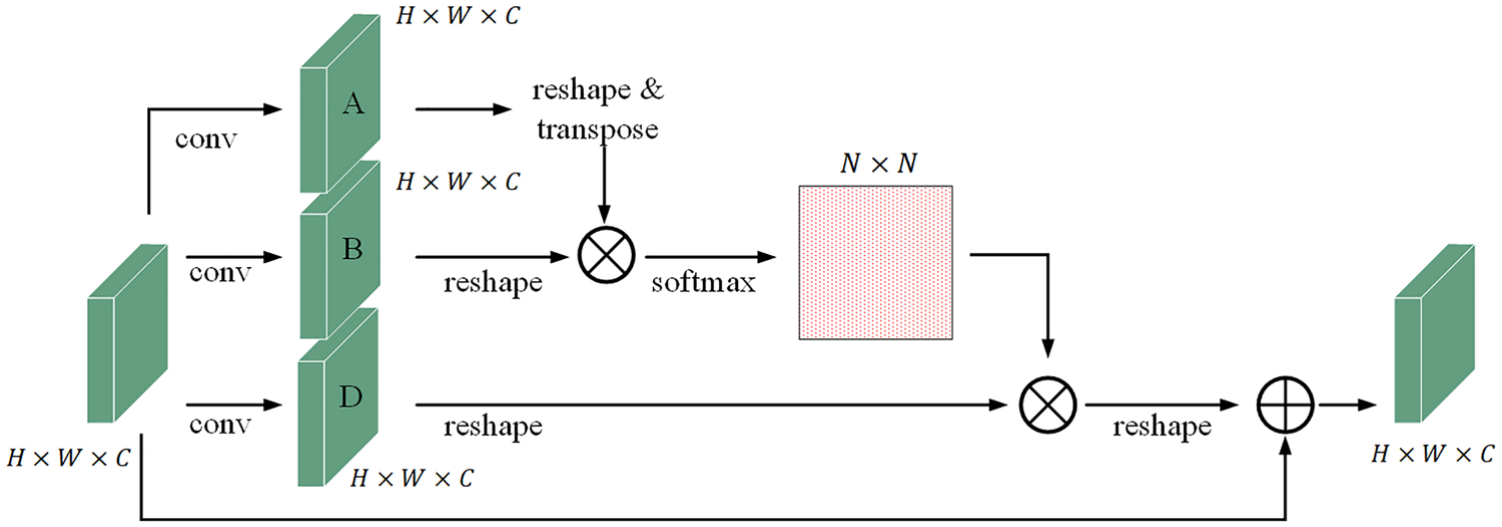
Detailed composition of the SAM.

The SAM (Fig. 5) consists of four steps. First, convolution operations on the input are implemented to obtain three feature maps: A, B, and C. The sizes of A, B, and D are H × W × C (where H represents height, W represents the width, and C represents the number of channels). Then the shape of A is changed into N × C (N = H × W) through reshaping and transposing, and the size of B is changed into C × N through reshaping. Matrix multiplication and Softmax activation on A and B are implemented to obtain the attention weight map of size N × N. The third step is to change the shape of D to C × N by reshaping and performing matrix multiplication with the attention weight map obtained in the second step to obtain the optimized feature map. Finally, the original feature map and the optimized feature map are subjected to matrix addition to obtain the final output.

### Datasets description

Two datasets: ISPRS Potsdam Dataset (https://www.isprs.org/education/benchmarks/UrbanSemLab/2d-sem-label-potsdam.aspx) and CCF BDCI 2020 Dataset (https://www.datafountain.cn/competitions/475) are used to assess the effect of AFF-UNet. The Potsdam dataset depicts a typical historical metropolis. The Potsdam dataset is a public dataset, which includes 38 images. Each image size is 6000*6000, 4 channels (red, green, blue, near-infrared) orthophotos are used in our experiments. For training, 30 frames are utilized, and for testing, 8 frames (6_9,7_7,7_8,7_9,7_10,7_11,7_12,7_13,) are used. There are six classes in the dataset (impervious surface, building, low vegetation, tree, car, background). The images are cropped to 400*400 pixels, and 6750 patches for training and 1800 patches for testing are acquired.

The CCF BDCI 2020 remote sensing image dataset (abbreviated as BDCI dataset) was made by the official CCF. In the experiment, 145,981 images (red, green, and blue) were used, each with the size of 256*256. The data set contains various common land-cover classes: background, woodland, cultivated field, grassland, building, road and water. In the experiment, 130,000 patches were used for training and 15,981 patches were used for testing.

### Experimental setting details

The Adam optimizer was used to train all the CNNs in this study, and the initial learning rate was 1e-4. The size of the minibatch was eight. The loss function is cross entropy loss. We trained the network on a Linux server with a Sugon DCU accelerator and a Hygon C86 7185 CPU until the loss converged. The random access memory (RAM) was 128 GB in capacity. In addition, TensorFlow 2.2 was used to build deep learning models in the trials.

The performance of approaches is assessed using four evaluation metrics: overall accuracy (OA), per-class F1 score, average F1 score, and mean intersection over union (mIOU). The proportion of accurately marked pixels in the total pixels is represented by OA. The harmonic mean of precision and recall is applied to calculate the F1 score for a given class. The mean correlation between the actual and predicted outcomes at the class level is measured using mIOU.

5 $$Precision=\frac{TP}{TP+FP},$$

6 $$Recall= \frac{TP}{TP+FN},$$

7 $$\mathrm{F}1=2\times \frac{precision \times recall}{precision+recall},$$

8 $$\mathrm{IOU}=\frac{TP}{TP+FN+FP},$$

9 $$\mathrm{OA}=\frac{TP+TN}{TP+FP+TN+FN}.$$

## Experimental result and analysis

### Ablation studies

Ablation studies using the Potsdam were implemented to validate the effectiveness of the AFF-UNet. The effectiveness of DSC and AFAM, CACB, and SAM has been discussed separately. The results of all the ablation experiments are shown in Table 1. In this section, the quantitative and visual analyses of the results of the ablation experiments are performed.

**Table 1:** Evaluation metrics results of ablation experiments on the Potsdam dataset with different configurations of the network.

Model	CACB	AFAM	SAM	OA	mIOU
Unet				82.01	66.44
UNet + CACB	√			84.46	69.96
UNet + CACB + AFAM	√	√		85.13	71.24
AFF-UNet	√	√	√	85.4	71.44

OA and mIOU are both expressed in percentages.

### Quantitative comparison

The outcomes of the ablation experiments are shown in Table 1, with UNet serving as the baseline model for comparison. In the table, UNet + CACB represents the UNet model applying CACB to obtain the channel relation. UNet + CACB + AFAM represents the UNet model applying CACB and AFAM. AFF-UNet is the proposed model that further applies the SAM based on applying the CACB and the AFAM.

As shown in Table 1, the application of CACB resulted in a 2.45% rise in OA and a 3.52% increase in mIOU. The application of AFAM resulted in a 3.12% rise in OA and a 4.8% increase in mIOU. It can be seen that each module produced different degrees of improvement. Table 1 shows that the performance of AFF-UNet was optimal. In terms of OA, AFF-UNet presents an increase of 3.39% over UNet. In aspects of mIOU, AFF-UNet had a 5% advantage over UNet. This further proves that the AFF-UNet can fuse the different levels of feature maps effectively and obtain more accurate feature representations.

### Qualitative comparison

**Figure 6:**
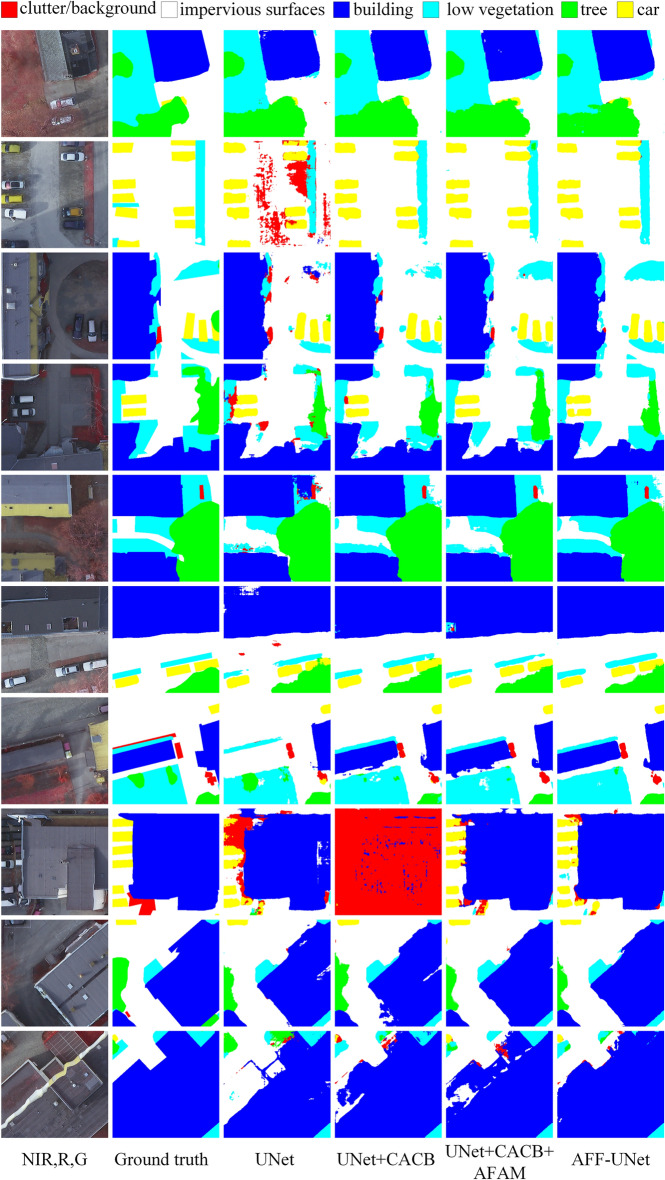
Visualization comparison of the ablation experiments on the Potsdam dataset.

**Figure 7:**
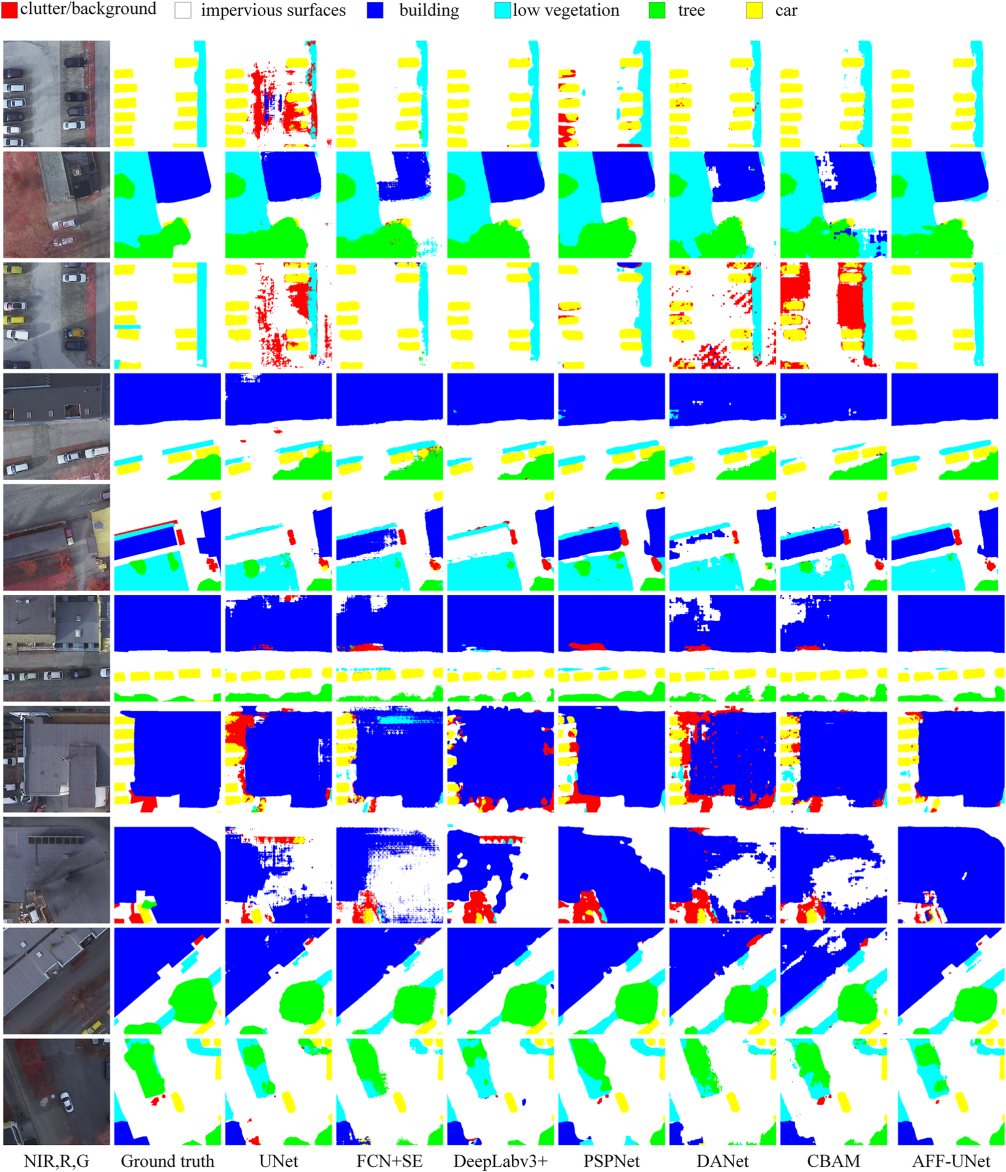
Visualization of the comparison experiments on the Potsdam dataset.

To visually assess if the designed modules were functional, the visualizations of the Potsdam dataset are shown in Figs. 6 and 7. In the ablation experiment results, the development of AFF-UNet can be summarized in the following two points:

1. The confusion of the object classes was reduced. The segmentation results of UNet in the third column of Fig. 6 show more phenomena of identifying non-background classes as background classes. Because the feature representation of the background class is complex, most geographic objects in the background class are meaningless. If the feature representation of the non-background class is inaccurate, it will be easy to misidentify the non-background class as the background class. After applying CACB, the confusion of the object classes was reduced to a certain extent, but it also provided an unstable performance (e.g. in row 8 and column 4, nearly the entire patch is misidentified as the back-ground class). This shows that only the channel attention mechanism was not enough. In our AFF-UNet, both the channel attention mechanism CACB and the SAM were applied, thus obtaining a more accurate feature representation with minimal class confusion in the segmentation results.
2. When there were different sizes of objects in the same patch, the proposed model had better performance. For example, in row 3, there are both large-size buildings, small-size vehicles, and low vegetation classes. Only AFF-UNet accurately segmented these three classes in row 3 at the same time. In Fig. 6, there are both buildings and vehicles in row 8, and the proposed model still segmented them well. This was due to the feature fusion ability produced by the DSC structure and the ability to distinguish different level feature blocks due to AFAM.

### Quantitative comparison with other models

The experimental comparison models are FCN + SE, DeepLabv3 + , PSPNet, DANet, and CBAM. The quantitative outcomes on the two datasets are shown in Tables 2 and 3, and UNet is still used as the baseline model. FCN + SE means applying the SE operation in FCN. From the results in Tables 2 and 3, the AFF-UNet achieved the best values of the comprehensive evaluation indicators average F1, OA, and mIOU.

**Table 2:** Quantitative comparisons of the Potsdam dataset with other models.

Method	Per-class F1 score					Avg. F1	OA	mIOU
	Impervious surface	Building	Low vegetation	Tree	Car			
UNet	85.88	88.14	73.19	78.9	85.13	82.25	82.01	66.44
FCN + SE	87.84	89.54	76.2	78.94	85.47	83.6	84.09	69.47
DeepLabV3 +	87.66	91.11	75.96	77.32	86.5	83.71	84.41	70.08
PSPNet	87.34	91	74.16	79.3	83.25	83.01	84.08	69.27
DANet	85.89	87.5	75.98	79.08	86.69	83.02	82.34	67.27
CBAM	86.98	88.94	76.14	76.94	86.42	83.08	83.22	68.44
**Proposed**	**88.81**	**92.03**	**76.89**	**79.31**	**86.96**	**84.8**	**85.4**	**71.44**

Significant values are in bold.

**Table 3:** Quantitative comparisons of the BDCI dataset with other models.

Method	Per-class F1 score						Avg. F1	OA	mIOU
	Woodland	Cultivated field	Grassland	Building	Road	Water			
Unet	90.14	92.28	18.82	79.61	40.82	92.54	69.04	88.59	62.36
FCN + SE	90.68	92.7	19.59	**93.94**	36.06	93.63	71.1	89.74	62.18
DeepLabV3 +	89.62	92.72	31.17	84.86	39.38	93.33	71.85	89.53	65.47
PSPnet	90.18	92.96	28.92	84.28	36.11	93.24	70.95	89.99	65.36
DAnet	90.79	93.18	28.35	84.65	47.7	94.28	73.16	90.33	67.31
CBAM	90.81	93.21	31.02	85.66	47.98	94.33	73.84	90.33	67.89
**Proposed**	**91.96**	**94.05**	**37.83**	86.45	**52.66**	**95.09**	**76.34**	**91.65**	**70.5**

Significant values are in bold.

The comparisons of the Potsdam are in Table 2. Among these comparison models, the worst performer was the baseline model UNet, and the best performer was DeepLabV3 + . The AFF-Unet outperformed all of the comparison models, with an average F1 of 84.8%, an OA of 85.4%, and a mIOU of 71.44%. With respect to the average, F1, the AFF-UNet was 2.55% better than UNet and 1.09% better than DeepLabV3 + . With respect to the OA, the AFF-UNet was 3.39% better than UNet and 0.99% better than DeepLabV3 + . With respect to the mIOU, the AFF-UNet outperformed UNet by 5% and DeepLabV3 + by 1.36%. The results demonstrated that the AFF-UNet performed better than other models on the Potsdam dataset. Applying the SE operation in FCN produced a better performance than the UNet. This result confirms that the application of the SE operation can optimize the segmentation results. DeepLabV3 + was superior to PSPNet because DeepLabV3 + not only expanded the receptive field through the atrous convolution but also fused the con-textual information through skip connections. The performances of DANet and CBAM were similar, and both achieved better results than the baseline model due to the self-attention mechanism. In general, the proposed model achieved improvement in both the OA and mIOU, which indicates that the strategies in the proposed model are effective.

The AFF-UNet outperformed all of the comparison models in the F1 scores on all classes. A comparison between the classes showed that the tree class and the low vegetation class were more difficult to segment. The reason for this is that tree class and low vegetation class are easily confused without height information. Hence, all of the models per-formed poorly in these two classes. The AFF-UNet model obtained a slight advantage in these two classes compared to the other models.

The comparison test results of the BDCI are shown in Table 3. Among these comparison models, the worst performer was the baseline model UNet, and the best performer was CBAM. This was different from the results of Potsdam, and in the following, we attempt to explain why this occurred. The AFF-UNet achieved the optimal performance, with an average F1 of 76.34%, OA of 91.65%, and mIOU of 70.5%. With respect to the average F1, the AFF-UNet outperformed UNet and CBAM by 7.3% and 2.5%, respectively. With respect to the OA, the proposed model was 3.06% better than UNet and 1.32% better than CBAM. With respect to the mIOU, the proposed model was 8.14% better than UNet and 2.61% better than CBAM. As shown in Table 3, almost all models have relatively low segmentation accuracy for low vegetation and trees, which is due to the difficulty in distinguishing between these two classes without height information. The results on the BDCI dataset demonstrated that the AFF-UNet performed better than the other models.

In the BDCI dataset, compared with the other three classes (woodland, cultivated field, and water), the F1 scores of these three classes (grassland, building, and road) were relatively low. This means that the latter three classes were more difficult to identify, especially the grassland and road. The F1 score indicated that the proposed AFF-UNet had the highest score in five classes and the second-highest score in the building class.

Different from the Potsdam dataset, the models (DeepLabV3 + and PSPNet) did not achieve better performances on the BDCI dataset. The reason is that the image size in the BDCI dataset was small, and the size difference of the various classes was not obvious. Hence, increasing the receptive field did not bring significant improvement. However, the attention mechanism models (DANet and CBAM) still achieved better performance. In general, the AFF-UNet model achieved better semantic segmentation performance on the BDCI dataset.

### Qualitative comparison with the other models

The small-scale (Fig. 7 corresponds to Potsdam) visualization results are shown in this section. The comparison among the various models included 1. performance of the segmenting confused object classes; 2. performance of the segmenting different sizes of object classes; and 3. the integrity of objects.

Performance of the segmenting confused object classes: Due to the complexity of the background class features, they are easily confused with a non-background class. The primary object classes in the row 1 and row 3 patch in Fig. 7 are vehicles, impervious sur-faces, and low vegetation. UNet, DANet, CBAM, and PSPNet all output obvious false segmentations, misidentifying impervious surfaces or vehicles as the background class. Among these two rows, FCN + SE, DeepLabV3 + , and the proposed model all displayed good performances.

The performance of segmenting different sizes of object classes: The proposed model more effectively considered the segmentation accuracy of large-size objects and small-size objects. For example, the patch in row 7 of Fig. 7 contains a large-size building and several small-size vehicles. All the comparison models were unable to consider the segmentation accuracy of both buildings and vehicles. The proposed model was able to segment different sizes of objects more effectively and obtained the most similar results to the ground truth.

Integrity of objects: As shown in Fig. 7, the integrity of the object class was better maintained by AFF-UNet, and the improvement in the integrity of the building class was especially obvious. In row 2 of Fig. 7, FCN + SE, DANet, and CBAM produced obvious errors in the segmentation of buildings, and they identified the middle portion of buildings as impervious surfaces, which is meaningless. A similar situation occurred in rows 4, 6, and 8, where these comparison models showed more fragmented results. In row 8, there was a building object that was difficult to segment, and only the proposed model obtained results close to the ground truth.

In general, our model demonstrated improved performance over the other comparison model, which can be attributed to the following improvements:(1) The proposed model has dense skip connections (DSC) between the encoder and decoder, which enhances the model's feature fusion ability and helps to obtain more accurate feature representations, thus improving classification accuracy. (2) The model uses channel attention convolutional blocks (CACB) as the basic building unit. By modeling the feature channels through convolution, activation, and channel attention mechanisms, the model enhances or suppresses different features to improve segmentation performance. (3) The adaptive fusion attention module (AFAM) distinguishes feature map blocks of different levels, enabling more flexible feature fusion and better adaptation to segment objects of different sizes simultaneously. (4) The spatial attention module (SAM) is placed between the encoder and decoder allows the model to perform attention operations on different positions of the feature map, further improving the segmentation accuracy of the model.

### Model complexity and the stability of the training process

**Figure 8:**
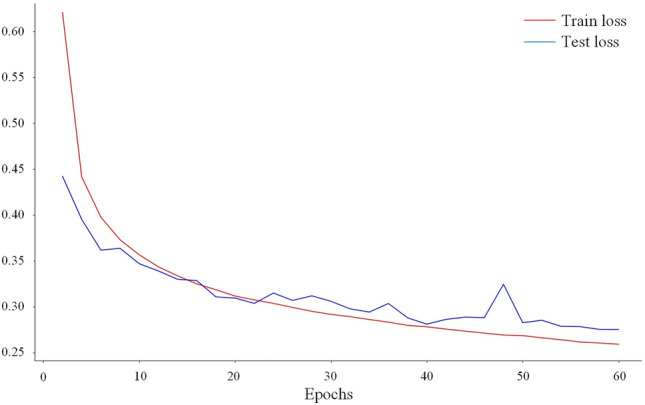
The loss value trend on the BDCI dataset.

In our experiments, with the same hardware environment and the same amount of training data, the proposed model took about 1.5 times longer to train than the original UNet model. However, our model achieved improvements in three aspects: (1) reduced category confusion; (2) improved segmentation results for different sizes of targets; and (3) improved target integrity. Regarding the stability of the training process, (1) in the model comparison experiments, we trained all models for the same number of epochs and selected the best performing epoch as our final output; (2) Adam optimizer was used during the training process, and the loss value trend on the BDCI dataset is shown in Fig. 8, indicating that the model trained relatively quickly and the process was stable and controllable.

## Conclusions

In this study, we proposed the AFF-UNet, which exhibits better performance in handling confusion between object classes and segmentation of different sizes of object classes, particularly buildings and vehicles, in RSI. To achieve this, we utilized a tailored CACB to obtain the channel relationship. We performed context aggregation and obtained the relationship between different levels of feature blocks using DSC structures and AFAM. Moreover, we utilized SAM to further improve segmentation accuracy by obtaining the relationship between different positions. The AFF-UNet was evaluated on two datasets, and compared with other models, it demonstrated improvements in (1) the confusion of the object classes; (2) achieving better segmentation results for different sizes of object classes; and (3) improving object class integrity. Our proposed model has potential to optimize binary classification tasks, such as extracting vehicles or buildings. However, the class imbalance in RSI segmentation datasets typically negatively impacts performance, and resolving this will be the focus of future work.

## Data Availability

The datasets used or analyzed during the current study are available from the corresponding author on reasonable request.
